# Fast Collisional Lipid Transfer Among Polymer-Bounded Nanodiscs

**DOI:** 10.1038/srep45875

**Published:** 2017-04-05

**Authors:** Rodrigo Cuevas Arenas, Bartholomäus Danielczak, Anne Martel, Lionel Porcar, Cécile Breyton, Christine Ebel, Sandro Keller

**Affiliations:** 1 Molecular Biophysics, University of Kaiserslautern, Erwin-Schrödinger-Str. 13, 67663 Kaiserslautern, Germany; 2 Institut Max von Laue Paul Langevin, Grenoble, 38042, France; 3 Institut de Biologie Structurale (IBS), Univ. Grenoble Alpes, CEA, CNRS, Grenoble, 38044, France

## Abstract

Some styrene/maleic acid (SMA) copolymers solubilise membrane lipids and proteins to form polymer-bounded nanodiscs termed SMA/lipid particles (SMALPs). Although SMALPs preserve a lipid-bilayer core, they appear to be more dynamic than other membrane mimics. We used time-resolved Förster resonance energy transfer and small-angle neutron scattering to determine the kinetics and the mechanisms of phospholipid transfer among SMALPs. In contrast with vesicles or protein-bounded nanodiscs, SMALPs exchange lipids not only by monomer diffusion but also by fast collisional transfer. Under typical experimental conditions, lipid exchange occurs within seconds in the case of SMALPs but takes minutes to days in the other bilayer particles. The diffusional and second-order collisional exchange rate constants for SMALPs at 30 °C are *k*_dif_ = 0.287 s^−1^ and *k*_col_ = 222 M^−1^s^−1^, respectively. Together with the fast kinetics, the observed invariability of the rate constants with probe hydrophobicity and the moderate activation enthalpy of ~70 kJ mol^−1^ imply that lipids exchange through a “hydrocarbon continuum” enabled by the flexible nature of the SMA belt surrounding the lipid-bilayer core. Owing to their fast lipid-exchange kinetics, SMALPs represent highly dynamic equilibrium rather than kinetically trapped membrane mimics, which has important implications for studying protein/lipid interactions in polymer-bounded nanodiscs.

Several styrene/maleic acid (SMA) copolymers solubilise membrane proteins and surrounding lipids to form SMA/lipid particles (SMALPs) without requiring conventional detergents^1^,^2^. SMALPs are disc-shaped nanoparticles with typical diameters of 10–25 nm^3^,^4^ that are made up of a lipid-bilayer patch bounded by a polymer belt^5^,^6^. Thus, their colloidal morphology is similar to that of lipid nanodiscs surrounded by amphipathic membrane scaffold proteins (MSPs)^7^. SMALPs are attracting great attention as a new membrane mimic because they can solubilise proteins from artificial^1^,^8^ or natural^9^,^10^,^11^,^12^,^13^ membranes while retaining a native-like environment in the form of a nanosized lipid bilayer. Owing to their small size, SMALPs are well suited for optical-spectroscopic^1^,^3^,^4^,^8^,^9^,^12^,^14^,^15^ and chromatographic^13^,^15^,^16^,^17^ techniques. Likewise, a diisobutylene/maleic acid (DIBMA) copolymer has been found^18^ to solubilise membrane proteins and lipids in a mild fashion, that is, without major perturbation of the bilayer order in DIBMA/lipid particles (DIBMALPs).

Different lines of evidence indicate that polymer-bounded nanodiscs present a more dynamic lipid environment than other bilayer-containing membrane-mimetic systems such as MSP nanodiscs. For instance, Dörr *et al*.^14^ have shown that single copies of an ion-channel protein can spontaneously transit from SMALPs to free-standing lipid bilayers. Very recently, Broecker *et al*.^19^ have demonstrated that a membrane protein can readily be transferred from SMALPs to lipidic cubic phases (LCPs) in amounts sufficient for growing well-diffracting crystals. We have reported^4^ that phospholipids populating the SMALP core can interact and re-associate with large unilamellar vesicles (LUVs), and Edler and co-workers^20^ have proposed that SMALPs might be able to exchange lipids with monolayers.

In spite of the above circumstantial evidence, nothing is known about the kinetics and the underlying mechanism(s) of lipid exchange, although such quantitative and mechanistic insights are essential if one is to fully exploit the potential of the SMALP technology for investigating biological membrane components *in vitro*. Here, we quantify the kinetics and identify the mechanisms of lipid transfer among SMALPs formed from the zwitterionic phospholipid 1,2-dimyristoyl-*sn*-glycero-3-phosphocholine (DMPC) and an SMA copolymer having a 3:1 styrene/maleic acid molar ratio (SMA(3:1)). Our results demonstrate that, unlike MSP nanodiscs and LUVs, SMALPs are highly dynamic rather than kinetically trapped structures that rapidly exchange lipids, which has important implications for exploiting “native nanodiscs” in membrane-protein studies.

## Results and Discussion

### Time-resolved Förster resonance energy transfer (TR-FRET)

Generally, the two major mechanisms of lipid transfer among nanoparticles are (i) desorption and interparticle diffusion of lipid monomers through the aqueous phase, which manifests in a saturable kinetic model^21^,^22^,^23^ (equation (1) in Supplementary Information), and (ii) lipid exchange through particle collisions, which gives rise to a second-order kinetic scenario (equation (2))^24^,^25^,^26^,^27^. When both mechanisms are at play, the observed rate constant of lipid exchange, *k*_obs_, contains contributions from both processes (equation (3)). We utilised time-resolved Förster resonance energy transfer (TR-FRET)^22^,^23^ to quantify the concentration dependence of *k*_obs_. To this end, we prepared SMALPs that contained 1 mol% of each of the two fluorescently labelled phospholipids *N*-(7-nitrobenz-2-oxa-1,3-diazol-4-yl)-1,2-dihexadecanoyl-*sn*-glycero-3-phosphoethanolamine (NBD-PE) and *N*-(lissamine rhodamine B sulphonyl)-1,2-dihexadecanoyl-*sn*-glycero-3-phosphoethanolamine (Rh-PE) in a DMPC matrix. With a Förster distance of *R*_0_ = 6.6 nm^28^, NBD-PE and Rh-PE form an efficient FRET pair when co-localised in the same SMALP, but the FRET efficiency should decrease when the two fluorescent lipids become diluted in a DMPC background.

**Figure 1:**
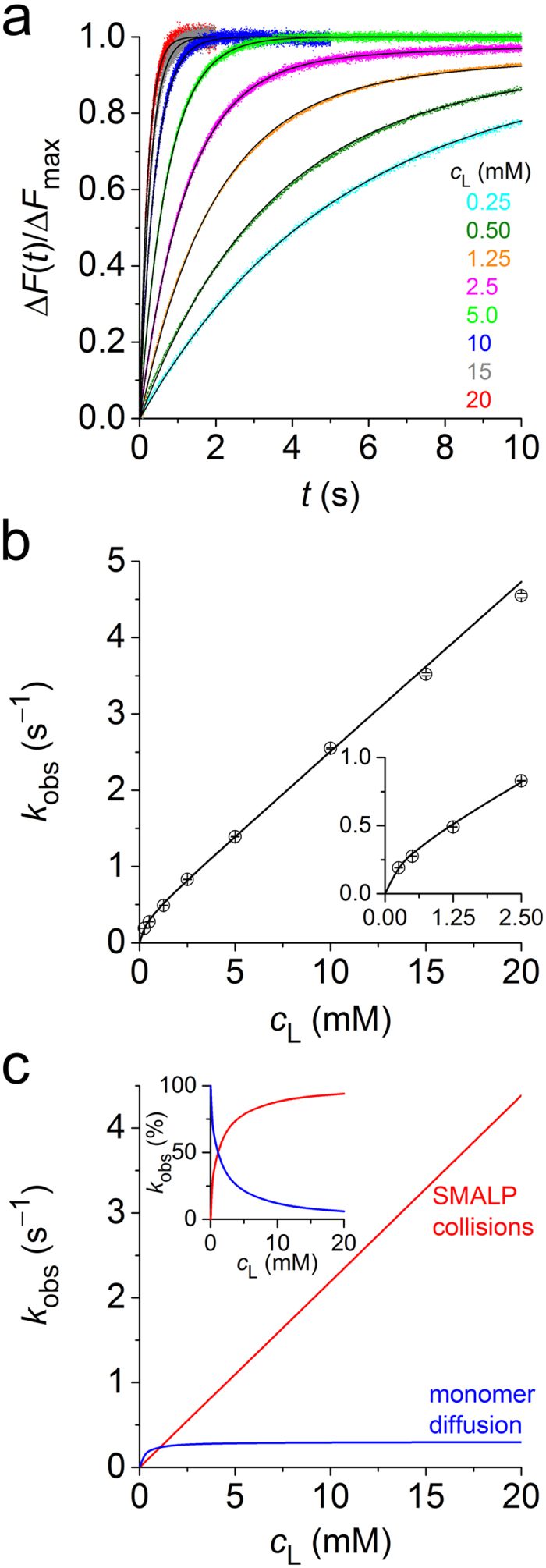
Transfer of fluorescently labelled lipids as monitored by TR-FRET at 30 °C. (**a**) Normalised NBD-PE fluorescence emission, Δ*F*(*t*)/Δ*F*_max_, *versus* time, *t*, upon mixing labelled SMALPs at a total lipid concentration of 0.25 mM with unlabelled SMALPs at various *c*_L_. Shown are experimental data (coloured dots) and a global fit (solid lines) based on equation (5). (**b**) Lipid exchange rate constants, *k*_obs_, derived from local fits to data in panel a according to equation (4) (open circles) and results from a global fit (solid line) according to equation (5). Error bars are 95% confidence intervals of local fits. *Inset*: Zoomed view at low *c*_L_. (**c**) Absolute contributions to *k*_obs_ of diffusional and collisional processes. *Inset*: Same data as relative contributions.

Indeed, we observed fast dequenching of the NBD-PE donor when mixing labelled SMALPs with various concentrations of unlabelled SMALPs in a stopped-flow apparatus (Fig. 1a). Local fits (equation (4)) yielded *k*_obs_ values across the entire concentration range studied (Fig. 1b). The steep increase in *k*_obs_ with the concentration of lipid in unlabelled SMALPs, *c*_L_, was linear at high *c*_L_ but showed pronounced downward curvature at low *c*_L_. Hence, the data could not be fitted on the assumption of a single mechanism (*i.e.*, either equation (1) or equation (2)) but demonstrated that both monomer diffusion and collisional exchange (equation (3)) were at play. A global fit (equation (5)) furnished best-fit values and 95% confidence intervals of the diffusional and second-order collisional exchange rate constants of, respectively, *k*_dif_ = (0.287 ± 0.005) s^−1^ and *k*_col_ = (222 ± 1) M^−1^s^−1^ at 30 °C. Plotting the contributions of diffusional and collisional transfer against *c*_L_ reveals that the latter dominates at all except the lowest *c*_L_ values (Fig. 1c). At *c*_L_ = 10 mM, for instance, collisional transfer accounts for >93% of the total exchange.

### Time-resolved small-angle neutron scattering (TR-SANS)

**Figure 2:**
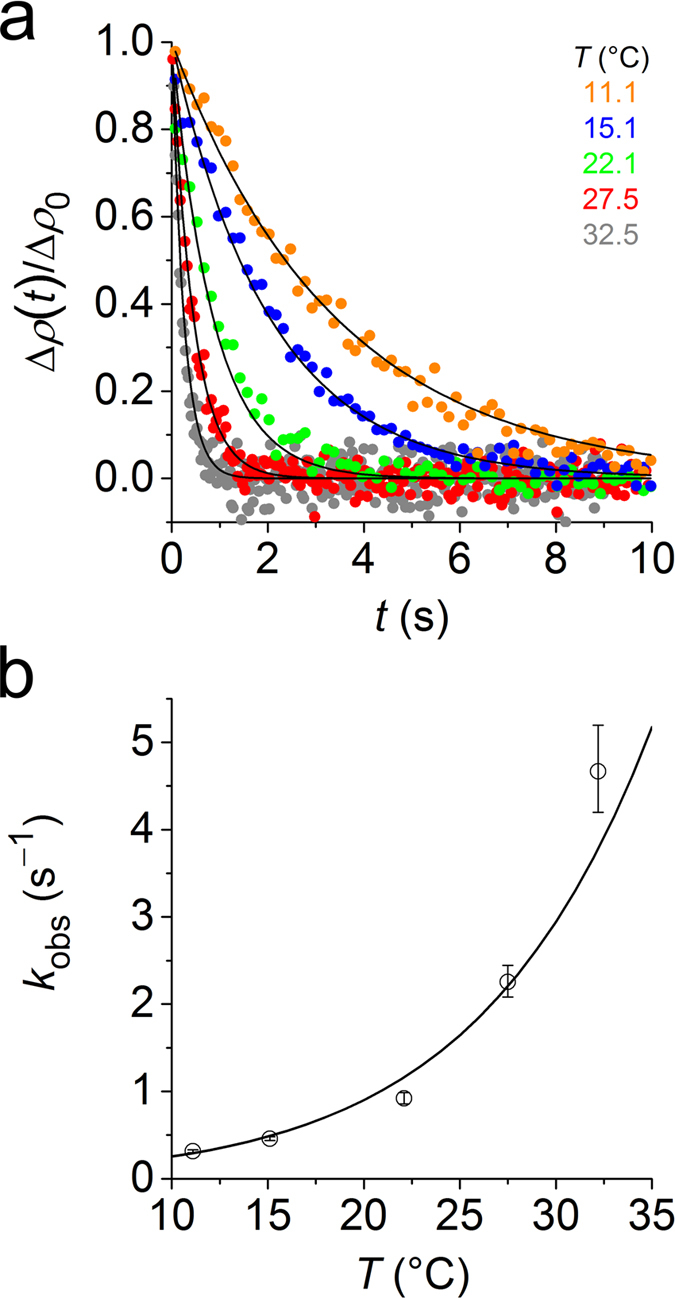
Transfer of DMPC among SMALPs as monitored by TR-SANS at various temperatures. (**a**) Decay of normalised SLD, ∆*ρ*(*t*)/∆*ρ*_0_, with time, *t*, upon mixing d- and h-SMALPs at 10 mM of each lipid and different temperatures, *T*. Shown are experimental data (coloured dots) and a global fit (solid lines) according to equation (8). (**b**) Lipid exchange rate constants, *k*_obs_, derived from local fits to data in panel a according to equation (7) and results from a global fit (solid line) according to equation (8). Error bars are 95% confidence intervals of local fits.

To shed further light on the lipid-exchange mechanism, we turned to time-resolved small-angle neutron scattering (TR-SANS), which does not depend on fluorescent labels. Instead, TR-SANS relies on monitoring changes in the neutron scattering length density (SLD) accompanying the exchange of molecules between hydrogenated and deuterated particles. This principle has been used to follow content exchange among copolymer micelles^29^,^30^ and, specifically, lipid transfer among MSP nanodiscs^31^ or LUVs^32^. We prepared SMALPs that harboured either hydrogenated or deuterated DMPC (h- and d-SMALPs, respectively) in Tris buffer containing 42.8% (*v*/*v*) D_2_O, which matches the SLD of nanodiscs composed of equal amounts of h- and d-DMPC (h/d-SMALPs). We then mixed equal volumes of the two SMALP preparations to reach a final concentration of 10 mM of each of the two lipids. The initially strong total SLD resulting from coexisting populations of h- and d-SMALPs rapidly decayed after mixing, with a monotonic decrease in decay time with increasing temperature (Fig. 2a). Since SMA(3:1) has been found^5^,^18^ to lower the gel-to-fluid transition temperature of DMPC, the lipid bilayers in the SMALP core were always in the fluid state, as confirmed calorimetrically (Supplementary Fig. 1). Each decay was fitted individually to yield temperature-dependent *k*_obs_ values (equation (6)). On raising the temperature from 11 °C to 33 °C, we thus observed a tenfold increase in *k*_obs_ (Fig. 2b). Interpolation to 30 °C yielded *k*_obs_ = (2.94 ± 0.29) s^−1^, which is in very good agreement with the TR-FRET data at *c*_L_ = 10 mM (Fig. 1b). Hence, although the two techniques are sensitive to fundamentally different physicochemical properties of two distinct lipid probes, they report on the same overall event, namely, inter-SMALP exchange of phospholipids.

**Figure 3:**
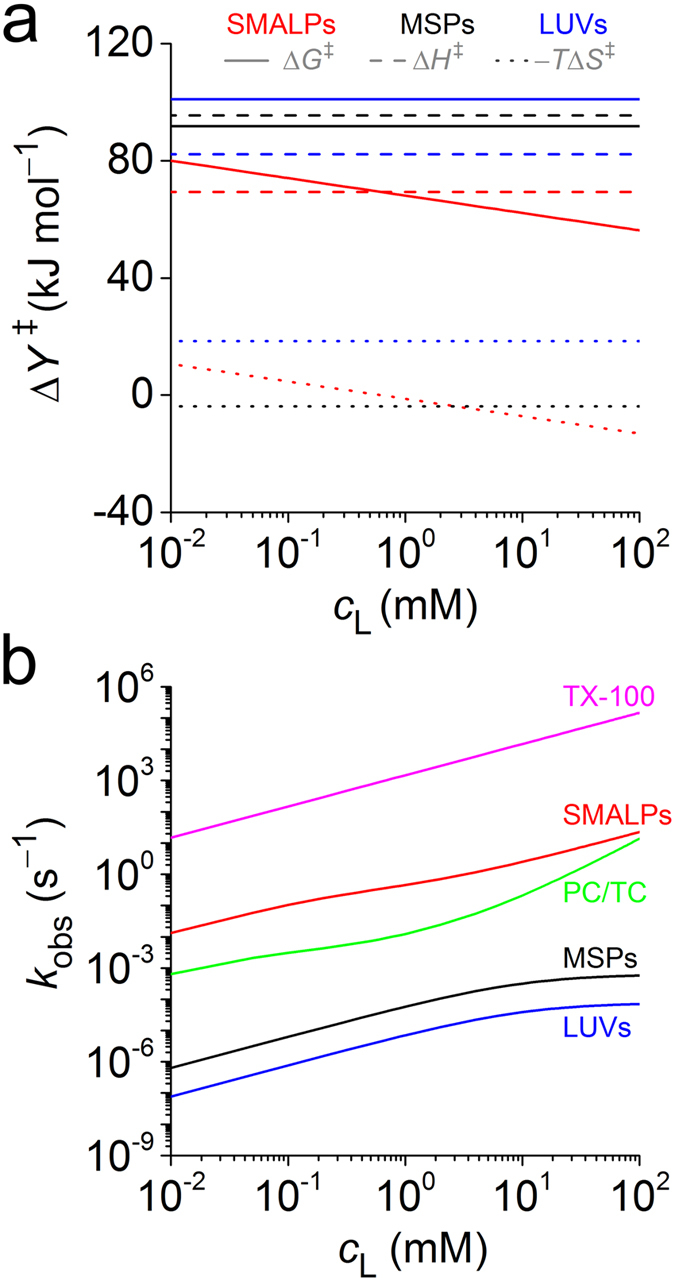
Kinetics of lipid exchange in different fluid-phase model membranes. (**a**) Changes in molar quantities of activation, Δ*Y*^‡^, at 37 °C for the Gibbs free energy, Δ*G*^‡^, enthalpy, Δ*H*^‡^, and entropy, −*T*Δ*S*^‡^, as obtained from equations (8) and (9) and data in Supplementary Table 1. (**b**) Lipid-exchange rate constants, *k*_obs_, as computed on the basis of equation (3) and data in Supplementary Table 2.

Since collisional lipid exchange dominates at *c*_L_ = 10 mM (see above), monomer diffusion can be neglected to a very good approximation. Thus, the TR-SANS results can be interpreted in terms of a collisional process with a second-order rate constant given by *k*_col_ ≈ *k*_obs_/*c*_L_ (equation (3)). Using a global fit based on transition-state theory^33^ (equations (7) and (8)), we analysed the temperature dependence of *k*_col_ to characterise the collisional transition state (Fig. 3a). This allowed a comparison with MSP nanodiscs^31^ and LUVs^32^, which exhibit lipid transfer solely by monomer diffusion. For both of the latter, the pronounced enthalpic penalty incurred upon formation of the transition state can be ascribed to the disruption of dispersive acyl-chain and polar headgroup interactions^26^,^27^. In agreement with this interpretation, Δ*H*^‡^ is substantially lower for SMALPs. While the entropic term is marginally favourable for MSP nanodiscs, it is moderately unfavourable for LUVs. For a second-order collisional process, −*T*Δ*S*^‡^ obviously depends on *c*_L_ (equation (9)). Within the experimentally relevant *c*_L_ range, we found the −*T*Δ*S*^‡^ values of SMALPs to be small in magnitude and, thus, similar to that of MSP nanodiscs and more favourable than that of LUVs. Consequently, Δ*G*^‡^ is more favourable for SMALPs than for either of the other two membrane mimics.

### Comparison of lipid-exchange kinetics and mechanisms in membrane mimics

We compiled *k*_obs_ values for various membrane mimics as calculated from published diffusional and collisional rate constants (Fig. 3b). At high *c*_L_, *k*_obs_ plateaus for monomer diffusion among MSP nanodiscs^31^ and LUVs^32^, whereas collisional exchange among SMALPs, small unilamellar vesicles (SUVs)^25^, phosphocholine/taurocholate (PC/TC) mixed micelles^24^, and Triton X-100 (TX-100) micelles^34^ leads to a steady rise in *k*_obs_ with *c*_L_. For PC/TC micelles, where transfer takes place through a combination of monomer diffusion and second-order (“bimolecular”) as well as third-order (“termolecular”) collisions, *k*_obs_ displays a particularly pronounced increase at high *c*_L_. At an exemplary concentration of *c*_L_ = 10 mM, lipid transfer is faster among SMALPs (*k*_obs_ = 2.50 s^−1^) than MSP nanodiscs (*k*_obs_ = 3.15 × 10^−4^ s^−1^), LUVs (*k*_obs_ = 3.83 × 10^−5^ s^−1^), SUVs (*k*_obs_ = 1.50 × 10^−4^ s^−1^), and even PC/TC micelles (*k*_obs_ = 0.211 s^−1^). Even faster kinetics have been reported for TX-100 micelles (*k*_obs_ = 1.5 × 10^5^ s^−1^). By contrast, DMPC exchange among gel-phase bicelles^35^ is slower than lipid transfer in any of the above fluid-phase systems.

The fast exchange kinetics among SMALPs results from higher *k*_dif_ and *k*_col_ values as compared with other systems, apart from TX-100 micelles (Supplementary Table 2). A high *k*_dif_ implies that lipid monomers can easily dissociate from SMALPs into the aqueous phase^21^,^22^,^23^, while a high *k*_col_ shows that lipids readily exchange during SMALP/SMALP collisions. It is instructive to relate *k*_col_ to the highest possible value expected for collisional exchange limited only by SMALP diffusion. Under the conditions used in this study, each ~24-nm SMALP contains ~1200 phospholipid molecules^4^. Therefore, *k*_col_, which refers to the *concentration of lipid monomers*, can be converted to the corresponding second-order rate constant referring to the *concentration of SMALP particles* according to *k’*_col_ = 1200^2^ × *k*_col_ = 3.2 × 10^8^ M^−1^s^−1^. At 30 °C, the diffusion-limited rate constant for collisions among nonionic particles amounts to *k*_col,max_ = 8.2 × 10^9^ M^−1^s^−1^ (equation (10))^34^. Thus, the efficiency of lipid exchange between two colliding SMALPs is ~4%, which is two and four orders of magnitude greater than the values reported for TX-100 micelles^34^ and PC/TC micelles^24^, respectively. Because charge repulsion must drastically decrease *k*_col,max_ in the case of polyanionic SMALPs, the actual efficiency of inter-SMALP lipid exchange will be even higher than the above crude estimate.

Collisional lipid exchange can, in principle, occur either through transient fusion of two lipid-containing particles to form a common hydrophobic core within which lipid molecules can move or, alternatively, through adhesion of two particles accompanied by lipid transfer across a (partially) hydrated polar layer^24^,^34^. For inter-SMALP lipid transfer, we favour the “hydrocarbon continuum” over the “sticky collision” model for three reasons: First, the fast kinetics resulting from the extraordinary efficiency of lipid transfer is more readily rationalised by a mechanism that allows rapid transfer *en masse*. Second, the similarity of the rate constants obtained from TR-FRET and TR-SANS suggests that the difference in hydrophobicity between the respective lipid probes plays no important role, which speaks against transfer across a polar medium. Third, the reduced enthalpic penalty upon formation of the transition state implies only moderate disruption of lipid/lipid interactions^26^,^27^. We hypothesise that the flexible nature of SMA^36^ enables fast reorganisation of the polymer belt upon collision of two SMALPs. This contrasts with the stiffness of the protein belt surrounding MSP nanodiscs^7^, which therefore exchange lipids only by much slower monomer diffusion^31^. For SUVs, collisional exchange prevails at high lipid concentrations but is thought to be based on the “sticky collision” mechanism, that is, monomer diffusion across a thin slab of aqueous phase separating the SUVs in the transition state^25^,^37^,^38^. Although substantial differences are seen between anionic PC/TC micelles^24^ and nonionic TX-100 micelles^34^, their small size, low aggregation number, and disordered, non-bilayer architecture endow micelles with inherently fast exchange kinetics, most likely mediated by a “hydrocarbon continuum”^34^.

Their highly dynamic lipid-exchange behaviour sets SMALPs apart from other bilayer-based membrane mimics such as MSP nanodiscs and LUVs, which has profound implications for the interpretation of membrane-protein studies relying on the unique properties of SMA. On the one hand, it has been found^14^,^39^ that protein-containing SMALPs isolated from native membranes are enriched in certain lipid species as compared with protein-free SMALPs. The present finding that SMALPs quickly exchange lipids then means that this enrichment must result from preferential protein/lipid interactions that are preserved in SMALPs, where they are sufficiently strong to retain these lipids in the immediate vicinity of the protein. In other words, the SMALPed “lipidome” is not merely a snapshot^40^ of the situation in the original membrane at the time of solubilisation but reflects rather strong protein/lipid interactions. Conversely, it seems plausible that weaker protein/lipid interactions, although present in the membrane before solubilisation, could be lost even after SMALP formation. On the other hand, an exciting perspective offered by fast interparticle lipid exchange lies in the possibility of systematically investigating the effects of various lipids on the structures, dynamics, and functions of SMALPed membrane proteins, which can easily be accomplished by changing the overall lipid composition through addition of SMALPs containing other types of lipids.

## Experimental

### Materials

1,2-dimyristoyl-*sn*-glycero-3-phosphocholine (h-DMPC) was obtained from Lipoid (Ludwigshafen, Germany), acyl-chain deuterated 1,2-dimyristoyl-*d*_54_-*sn*-glycero-3-phosphocholine (d-DMPC) from Cortecnet (Paris, France), *N*-(7-nitrobenz-2-oxa-1,3-diazol-4-yl)-1,2-dihexadecanoyl-*sn*-glycero-3-phosphoethanolamine (NBD-PE) from Fisher Scientific (Schwerte, Germany), and *N*-(lissamine rhodamine B sulphonyl)-1,2-dihexadecanoyl-*sn*-glycero-3-phosphoethanolamine (Rh-PE) from Biotium (Fremont, USA). SMA(3:1) copolymer solution (trade name XIRAN SL25010 S25) was a kind gift from Polyscope (Geleen, Netherlands). D_2_O was from Deutero (Kastellaun, Germany), chloroform from Fisher Scientific, NaCl from VWR (Darmstadt, Germany), and tris(hydroxymethyl)aminomethane (Tris) from Carl Roth (Karlsruhe, Germany). All chemicals were obtained in the highest purity available.

### Preparation of SMA(3:1) stock solution

SMA(3:1) is a random copolymer with a styrene/maleic acid molar ratio of 3:1 and mass- and number-average molar masses of *M*_*w*_ = 10 kg/mol and *M*_*n*_ = 4 kg/mol, respectively. Stock solutions of SMA(3:1) were prepared as described previously^3^,^4^. Briefly, 3 mL of a commercial XIRAN SL25010 S25 solution was dialysed against buffer (50 mM Tris, 200 mM NaCl, pH 7.4) using a 5-mL QuixSep dialyser (Membrane Filtration Products, Seguin, USA) and a Spectra/Por 3 dialysis membrane (Spectrum Laboratories, Rancho Dominguez, USA) with a molar-mass cut-off of 3.5 kg/mol. Dialysis was performed for 24 h under gentle stirring with membrane and buffer exchange after 16 h. Dialysed SMA(3:1) was filtered through a 0.22-μm poly(vinylidene fluoride) (PVDF) filter (Carl Roth, Karlsruhe, Germany). Final SMA(3:1) concentrations were determined by refractometry on an Abbemat 500 (Anton Paar, Graz, Austria). Molar concentrations were calculated from mass concentrations on the basis of the above number-average molar mass. Samples were stored at −20 °C.

### TR-FRET

Fluorescently labelled SMALPs were produced by suspending dry lipid powders in chloroform and mixing these solutions in a dark glass vial at 98:1:1 mol% of DMPC, NBD-PE, and Rh-PE, respectively. At this mixing ratio, NBD-PE emission (*λ*_ex_ = 475 nm, *λ*_em_ = 530 nm) is efficiently quenched by FRET to Rh-PE (*λ*_ex_ = 560 nm, *λ*_em_ = 582 nm)^41^. The lipid mixture was dried in a rotary evaporator at 60 °C and 20 kPa for 1 h and incubated in a desiccator at room temperature and 5 Pa for 16 h to remove traces of chloroform. The dry lipid film was suspended in 1 mL buffer (50 mM Tris, 200 mM NaCl, pH 7.4), and 10 freeze/thaw cycles were performed to produce multilamellar vesicles (MLVs). Labelled SMALPs were formed by incubating this suspension with SMA(3:1) at 30 °C for 16 h to yield final concentrations of 0.5 mM total lipid and 83 μM SMA(3:1). To produce unlabelled SMALPs, DMPC MLVs without fluorescent lipids were solubilised at 30 °C for 16 h to yield final concentrations of 40 mM DMPC and 6.6 mM SMA(3:1). These concentrations correspond to an SMA(3:1)/lipid molar ratio of 0.165, which is above the minimal ratio required for complete solubilisation^4^. To confirm complete solubilisation from hydrodynamic particle size distributions, dynamic light scattering (DLS) experiments were performed on a Zetasizer Nano S90 (Malvern Instruments, Malvern, UK) coupled to a 633-nm He–Ne laser and a photodetector mounted at an angle of 90°. Measurements were carried out in a 45-μL quartz glass cuvette with a 3 mm × 3 mm cross-section (Hellma Analytics, Müllheim, Germany) at 30 °C. These measurements yielded *z*-average particle sizes and associated size-distribution widths of (24 ± 12) nm for both labelled and unlabelled SMALPs.

TR-FRET was performed on an SF.3 stopped-flow apparatus (Applied Photophysics, Leatherhead, UK) coupled to a right-angle photomultiplier. NBD-PE was excited by a (470 ± 20) nm light-emitting diode (LED) with the current set to 20 mA using a 2.0-OD attenuator to prevent fluorophore bleaching. Fluorescence emission was blocked below 513 nm and above 543 nm with a TechSpec OD 6 band-pass filter with a centre wavelength of 527 nm (Edmund Optics, Karlsruhe, Germany). The drive syringes, tubes, and quartz cuvette were thermostated at 30 °C, and samples were equilibrated for at least 10 min prior to each measurement. 75-μL aliquots of unlabelled SMALPs at DMPC concentrations of 40, 30, 20, 10, 5, 2.5, 1.0, and 0.5 mM were mixed with equal volumes of labelled SMALPs harbouring 0.5 mM total lipid in a 20-μL quartz glass cuvette having a 2 mm × 2 mm cross-section. After each mixing step, at least 3000 data points were acquired with an integration time of 0.2–6 ms. At each concentration, a series of at least 10 traces were recorded and averaged for analysis by nonlinear least-squares fitting^42^.

### TR-SANS

h-SMALPs and d-SMALPs were produced by solubilising MLVs made from either h- or d-DMPC with SMA(3:1) at 30 °C for 16 h to yield final concentrations of 20 mM lipid and 3.3 mM SMA(3:1). Both d- and h-SMALPs were formed in buffer (50 mM Tris, 200 mM NaCl, pH 7.4) containing either 77.5% or 0% (*v*/*v*) D_2_O. Complete solubilisation was confirmed by DLS as described above.

TR-SANS measurements were performed on the D22 beam line at the Institut Laue–Langevin (Grenoble, France). The beam line is fed by a cold neutron source that emits monochromatic neutrons at *λ* = (6.0 ± 0.6) Å and is equipped with an SFM-300 stopped-flow apparatus (Biologic, Seyssinet-Pariset, France) with a 200-μL quartz glass cell having a pathlength of 1 mm. The instrument was set up for rectangular collimation of 40 mm × 55 mm at a sample/detector distance of 5.6 m and a sample aperture of 7 mm × 10 mm. The drive syringes, tubes, and quartz cuvette were temperature-controlled, and samples were equilibrated for at least 5 min prior to each measurement. The D_2_O concentration in the buffer was adjusted to 42.8% (*v*/*v*) to match the SLD contrast of SMALPs harbouring equal amounts of h- and d-DMPC. This condition was experimentally determined from static SANS measurement of h-SMALPs and d-SMALPs in 77.5% and 0% (*v*/*v*) D_2_O. 450-μL aliquots of d- and h-SMALPs in 42.8% (*v/v*) D_2_O were mixed at a total speed of 5 mL/s at 11.1, 15.1, 22.1, 27.5, and 32.5 °C. The dead time was estimated to be 3.1 ms. The full detector range intensity was integrated with an exposure time per data point of 0.15 s at 11.1 °C, 15.1 °C, and 22.1 °C; 0.05 s at 27.5 °C; and 0.03 s at 32.5 °C. SANS data (DOI: 10.5291/ILL-DATA.8-03-872) were collected using a 2D detector and reduced to 1D using the reduction package GRASP^43^. At each temperature, at least five traces were averaged and analysed by nonlinear least-squares fitting^42^.

## Additional Information

**How to cite this article:** Cuevas Arenas, R. *et al*. Fast Collisional Lipid Transfer Among Polymer-Bounded Nanodiscs. *Sci. Rep.* **7**, 45875; doi: 10.1038/srep45875 (2017).

**Publisher's note:** Springer Nature remains neutral with regard to jurisdictional claims in published maps and institutional affiliations.

## Supplementary Material

Supplementary Information
