# Supplementary material for: Fast Collisional Lipid Transfer Among Polymer-Bounded Nanodiscs
Source: Sci Rep. 2017 Apr 5;7:45875. doi: 10.1038/srep45875 (PMC5381093; doi:10.1038/srep45875)
Supplement: Supplementary Information [file srep45875-s1.pdf]

Supplementary Information

Fast Collisional Lipid Transfer Among Polymer-Bounded Nano-discs

Rodrigo Cuevas Arenas[1,#], Bartholomäus Danielczak[1,#], Anne Martel[2], Lionel Porcar[2], Cécile Breyton[3], Christine Ebel[3], Sandro Keller[1,*]

[1] Molecular Biophysics, University of Kaiserslautern, Erwin-Schrödinger-Str. 13, 67663 Kaiserslautern, Germany.

[2] Institut Max von Laue Paul Langevin, 38042 Grenoble, France.

[3] Institut de Biologie Structurale (IBS), Université Grenoble Alpes, CEA, CNRS, 38044 Grenoble, France.

* mail@sandrokeller.com

[#] These authors contributed equally to this work.

Theoretical Background 2

Supplementary Figure 5

Supplementary Tables 6

Supplementary References 8

Theoretical Background

Kinetics of phospholipid exchange among SMALPs

The transfer of lipid molecules among nanoparticles can take place through (i) desorption and interparticle diffusion of lipid monomers through the aqueous phase[1–3] and (ii) lipid exchange through particle collisions[4–7]. If the particles making up the two populations that exchange lipid molecules are identical in size and shape, the observed rate constant resulting from monomer diffusion takes the form[4,5,7]:

$$k_{\mathrm{obs,dif}}\,(c_{\mathrm{L}}) = \frac{k_{\mathrm{dif}} c_{\mathrm{L}}}{c_{\mathrm{L}}^{\circ} + c_{\mathrm{L}}} \quad (1)$$

where k_{dif} is the diffusional exchange rate constant and c_{L}° and c_{L} are the bulk solution concentrations of lipid in the donor and acceptor populations, respectively. For second-order ("bimolecular") collision-dependent lipid transfer, the observed rate constant reads:

$$k_{\mathrm{obs,col}}\,(c_{\mathrm{L}}) = k_{\mathrm{col}} c_{\mathrm{L}} \quad (2)$$

where k_{col} is the second-order collisional exchange rate constant. If both of the above processes are at play, the overall observed rate constant is given by the sum of equations (1) and (2)[4,5,7]:

$$k_{\mathrm{obs}}\,(c_{\mathrm{L}}) = \frac{k_{\mathrm{dif}} c_{\mathrm{L}}}{c_{\mathrm{L}}^{\circ} + c_{\mathrm{L}}} + k_{\mathrm{col}} c_{\mathrm{L}} \quad (3)$$

For SMALPs, including higher-order collisional events did not further improve the fit (Supplementary Table 1), suggesting that collisions among more than two SMALPs can be neglected. For example, third-order ("termolecular") collisional transfer would require an additional term proportional to ${c_{\mathrm{L}}}^2$ [4,5,7].

Concentration-dependent TR-FRET decays

Upon mixing fluorescently labelled and unlabelled SMALPs, NBD-PE and Rh-PE redistribute among all available SMALPs. This dilution of the fluorescent probe leads to dequenching of NBD-PE, which manifests in an exponential increase in the fluorescence emission intensity at 530 nm according to:

$$F(t) = F_{\infty} + e^{-k_{\mathrm{obs}} t}(F_0 - F_{\infty}) + mt \quad (4)$$

Here, $F(t)$ is the intensity at time t, F_0 and F_{∞} are the baseline-corrected original and final intensities, respectively, and m is the slope of the final baseline, which accounts for linear

signal drift at long times. For global data analysis, equation (3) was inserted into equation (4) to yield:

$$F(t) = F_\infty + e^{-\left(\frac{k_\mathrm{dif} c_\mathrm{L}}{c_\mathrm{L}^\circ + c_\mathrm{L}} + k_\mathrm{col} c_\mathrm{L}\right)t}(F_0 - F_\infty) + mt \tag{5}$$

In this global fitting equation, k_dif and k_col are global fitting parameters, whereas F_0, F_∞, and m are local (i.e., c_L-specific) fitting parameters. Best-fit parameter values and 95% confidence intervals were derived by nonlinear least-squares fitting in Excel spreadsheets as detailed elsewhere[8].

Temperature-dependent TR-SANS decays

Upon mixing h- and d-SMALPs, lipid exchange leads to an exponential decrease in the SLD with time, as given by:

$$I(t) = I_\infty + e^{-k_\mathrm{obs} t}(I_0 - I_\infty) \tag{6}$$

where $I(t)$ is the signal intensity at time t, I_0 and I_∞ are the initial and final intensities, respectively. According to transition-state theory[9], the second-order collisional rate constant can be expressed as a function of temperature, T, according to:

$$k_\mathrm{col}(T) = \frac{1}{\mathrm{M}} \frac{RT}{N_\mathrm{A} h} e^{-\Delta H^\ddagger/RT} e^{\Delta S^\ddagger/R} \tag{7}$$

with R being the universal gas constant, N_A Avogadro’s number, h Planck’s constant, and $\Delta H^\ddagger$ and $\Delta S^\ddagger$ the activation enthalpy and entropy, respectively. M denotes the unit “molar”, with c_L = 1 M being the standard concentration. At the c_L values used for TR-SANS, the relative contribution of monomer diffusion to the overall lipid transfer rate is very small (Fig. 1c), so that the first term on the right-hand side of equation (3) can be neglected. Thus, insertion of equation (7) into equation (3) and further into equation (6) yields:

$$I(t) = I_\infty + e^{-t\left((RT/N_\mathrm{A} h) e^{-\Delta H^\ddagger/RT} e^{\Delta S^\ddagger/R}\right) c_\mathrm{L}/\mathrm{M}}(I_0 - I_\infty) \tag{8}$$

In this global fitting equation, $\Delta H^\ddagger$ and $\Delta S^\ddagger$ are treated as global fitting parameters, whereas I_0 and I_∞ are local (i.e., T-specific) fitting parameters. Again, best-fit parameter values and 95% confidence intervals were derived by nonlinear least-squares fitting in Excel spreadsheets[8]. In the light of the broad temperature range across which phospholipids in SMALPs transition from the gel to the liquid–crystalline (i.e., fluid) state (Supplementary Figure 1), TR-SANS data were fitted in two ways, namely, once including and once excluding the data acquired at 11.1 °C. However, no significant differences were found in the best-fit values of $\Delta H^\ddagger$ or $\Delta S^\ddagger$.

The entropic component at arbitrary c_L values is related to the above standard-state value at c_L = 1 M through:

$$-T\Delta S^{\ddagger}(c_{\mathrm{L}}) = -T\Delta S^{\ddagger}(1\ \mathrm{M}) - RT\ln(c_{\mathrm{L}}/1\ \mathrm{M}) \quad (9)$$

The apparent values of the molar activation enthalpy and entropy were corrected for the temperature dependence of the buffer viscosity as detailed elsewhere[10].

Diffusion-limited collisional lipid transfer

In order to estimate the lipid-exchange efficiency of SMALP/SMALP collisions, we determined the diffusion-limited collisional rate constant that would be applicable to nonionic particles as[11]:

$$k_{\mathrm{col,max}} = \frac{8RT}{3\eta} \quad (10)$$

where η denotes the dynamic viscosity of the buffer.

Supplementary Figure

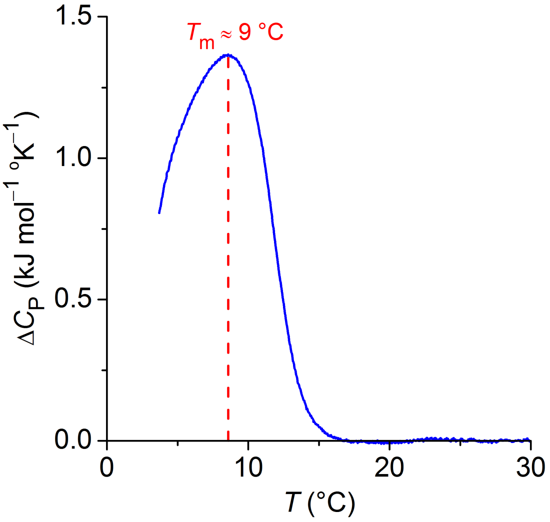

Supplementary Figure 1 Excess isobaric heat capacity, ΔC_p (blue solid line), *versus* temperature, T, for h/d-SMALPs at a total DMPC concentration of 10 mM as monitored by differential scanning calorimetry. The maximum ΔC_p value (red dashed line) was taken as T_m. The experiment was performed on a VP-DSC (Malvern Instruments, Worcestershire, UK). The sample and reference cells were filled with buffer (50 mM Tris, 200 mM NaCl, pH 7.4) and heated and cooled at a rate of 6 °C h^{-1}. Then, the content of the sample cell was replaced by a solution containing h/d-SMALPs. Data obtained from three up-scans were averaged, buffer- and baseline-subtracted, and normalised with respect to total lipid concentration using the software Origin 7.0 (OriginLab, Northampton, USA).

Supplementary Tables

Supplementary Table 1 Molar changes in Gibbs free energy of activation, $\Delta G^{\ddagger}$, enthalpy of activation, $\Delta H^{\ddagger}$, and entropy of activation, $-T\Delta S^{\ddagger}$, characterising phospholipid transfer among DMPC SMALPs, DMPC MSP nanodiscs, and DMPC LUVs at a reference temperature of 37 °C and, in the case of SMALPs, a reference lipid concentration of 10 mM, as determined by TR-SANS. 95% confidence intervals are indicated in parentheses below best-fit values.

**Bilayer system**	**$\Delta G^{\ddagger}$ (kJ mol^{-1})**	**$\Delta H^{\ddagger}$ (kJ mol^{-1})**	**$-T\Delta S^{\ddagger}$ (kJ mol^{-1})**	**Ref.**
DMPC SMALPs	60.4<br>(60.0–60.7)	69.4<br>(62.5–72.3)	−9.0<br>−(3.7–14.2)	*
DMPC MSP nanodiscs	91.8	95.5	−3.8	12
DMPC LUVs	101	82.2	18.4	13

* this work

Supplementary Table 2 Diffusional rate constants, k_{dif}, and second- and third-order collisional rate constants, $k_{col,2°}$ and $k_{col,3°}$, respectively, for the transfer of lipids among DMPC SMALPs, DMPC MSP nanodiscs, DMPC LUVs, DMPC SUVs, PC/TC mixed micelles, and TX-100 micelles. 95% confidence intervals are indicated in parentheses below best-fit values.

System	k_{dif} (s^{-1})	$k_{col,2°}$ ($M^{-1}s^{-1}$)	$k_{col,3°}$ ($M^{-2}s^{-1}$)	T (°C)	Ref.
DMPC SMALPs	0.287<br>(0.282–0.293)	222<br>(221–223)	–	30.0	*
DMPC MSP nanodiscs	6.3×10^{-4}	–	–	27.0	12
DMPC LUVs	7.7×10^{-5}	–	–	27.1	13
DMPC SUVs	1.13×10^{-4}<br>($\pm 1.39 \times 10^{-5}$)	3.6×10^{-3}<br>($\pm 8.3 \times 10^{-4}$)	–	30	6
PC/TC micelles	3.4×10^{-3}<br>($\pm 5.1 \times 10^{-4}$)	7.8<br>(±0.50)	1.3×10^{3}<br>(±85)	25	4
TX-100 micelles	12.6	1.47×10^{6}	–	24.6	14

* this work

Supplementary References

1. Nichols, J. W. & Pagano, R. E. Kinetics of soluble lipid monomer diffusion between vesicles. *Biochemistry* **20,** 2783–2789 (1981).
2. Nichols, J. W. & Pagano, R. E. Use of resonance energy transfer to study the kinetics of amphiphile transfer between vesicles. *Biochemistry* **21,** 1720–1726 (1982).
3. Nichols, J. W. Thermodynamics and kinetics of phospholipid monomer–vesicle interaction. *Biochemistry* **24,** 6390–6398 (1985).
4. Nichols, J. W. Phospholipid transfer between phosphatidylcholine–taurocholate mixed micelles. *Biochemistry* **27,** 3925–3931 (1988).
5. Fullington, D. A., Shoemaker, D. G. & Nichols, J. W. Characterization of phospholipid transfer between mixed phospholipid–bile salt micelles. *Biochemistry* **29,** 879–886 (1990).
6. Jones, J. D. & Thompson, T. E. Mechanism of spontaneous, concentration-dependent phospholipid transfer between bilayers. *Biochemistry* **29,** 1593–1600 (1990).
7. Fullington, D. A. & Nichols, J. W. Kinetic analysis of phospholipid exchange between phosphatidylcholine/taurocholate mixed micelles: Effect of the acyl chain moiety of the micellar phosphatidylcholine. *Biochemistry* **32,** 12678–12684 (1993).
8. Kemmer, G. & Keller, S. Nonlinear least-squares data fitting in Excel spreadsheets. *Nat. Protoc.* **5,** 267–281 (2010).
9. Fernández-Ramos, A., Miller, J. A., Klippsenstein, S. J. & Truhlar, D. G. Modeling the kinetics of bimolecular reactions. *Chem. Rev.* **106,** 4518–4584 (2006).
10. Perl, D., Jacob, M., Bánó, M., Stupák, M., Antalík, M. & Schmid, F. X. Thermodynamics of a diffusional protein folding reaction. *Biophys. Chem.* **96,** 173–190 (2002).
11. Kuriyan, J., Konforti, B. & Wemmer, D. in *The Molecules of Life. Physical and Chemical Principles* 815–817 (Garland Science, 2012).
12. Nakano, M. *et al.* Static and dynamic properties of phospholipid bilayer nanodiscs. *J. Am. Chem. Soc.* **131,** 8308–8312 (2009).
13. Nakano, M., Fukuda, M., Kudo, T., Endo, H. & Handa, T. Determination of interbilayer and transbilayer lipid transfer by time-resoved small-angle neutron scattering. *Phys. Rev. Lett.* **98,** 238101 (2007).
14. Rharbi, Y., Li, M., Winnik, M. A. & Hahn, K. G. Temperature dependence of fusion and fragmentation kinetics of Triton X-100 micelles. *J. Am. Chem. Soc.* **122,** 6242–6251 (2000).
